# Risk score model to automatically detect prostate cancer patients by integrating diagnostic parameters

**DOI:** 10.3389/fonc.2024.1323247

**Published:** 2024-05-15

**Authors:** Rossana Castaldo, Valentina Brancato, Carlo Cavaliere, Leandro Pecchia, Ester Illiano, Elisabetta Costantini, Alfonso Ragozzino, Marco Salvatore, Emanuele Nicolai, Monica Franzese

**Affiliations:** ^1^ Bioinformatics and Biostatistics Lab, IRCCS SYNLAB SDN, Naples, Italy; ^2^ School of Engineering, University of Warwick, Coventry, United Kingdom; ^3^ Università Campus Bio-Medico Roma, Roma, Italy; ^4^ Campus Bio-Medico, Fondazione Policlinico Universitario, Roma, Italy; ^5^ Adrology and Urogynecological Clinic, Santa Maria Terni Hospital, University of Perugia, Terni, Italy

**Keywords:** prostate cancer, MRI, machine learning, risk score, radiomics

## Abstract

**Introduction:**

Prostate cancer (PCa) is one of the prevailing forms of cancer among men. At present, multiparametric MRI is the imaging method for localizing tumors and staging cancer. Radiomics plays a key role and hold potential for PCa detection, reducing the need for unnecessary biopsies, characterizing tumor aggression, and overseeing PCa recurrence post-treatment.

**Methods:**

Furthermore, the integration of radiomics data with clinical and histopathological data can further enhance the understanding and management of PCa and decrease unnecessary transfers to specialized care for expensive and intrusive biopsies. Therefore, the aim of this study is to develop a risk model score to automatically detect PCa patients by integrating non-invasive diagnostic parameters (radiomics and Prostate-Specific Antigen levels) along with patient’s age.

**Results:**

The proposed approach was evaluated using a dataset of 189 PCa patients who underwent bi-parametric MRI from two centers. Elastic-Net Regularized Generalized Linear Model achieved 91% AUC to automatically detect PCa patients. The model risk score was also used to assess doubt cases of PCa at biopsy and then compared to bi-parametric PI-RADS v2.

**Discussion:**

This study explored the relative utility of a well-developed risk model by combining radiomics, Prostate-Specific Antigen levels and age for objective and accurate PCa risk stratification and supporting the process of making clinical decisions during follow up.

## 1 Introduction

Prostate cancer (PCa) is very common in elderly males worldwide (1). The benchmark for diagnosing PCa involves the histological examination of prostate tissue acquired through transrectal ultrasound-guided needle biopsy. The Gleason score is the prevalent scale utilized to assess the grade of PCa (2). Given that prostate cancers frequently consist of various malignant cells with differing grades, two grades are assigned for each case. The primary grade pertains to the most expansive cancerous area, while the secondary grade characterizes cells in the second largest area following the primary one. A prostate cancer diagnosis can be designated one of the following Gleason scores (based on its extent): a) 3 + 3 = 6: Cells exhibit a resemblance to healthy cells, indicating a well-differentiated state; b) 3 + 4 = 7: The cancer primarily comprises well-formed glands, but may contain a small portion of poorly formed, fused, or cribriform glands; c) 4 + 3 = 7: The cancer predominantly features poorly formed, fused, and/or cribriform glands with fewer well-formed glands; d) 4 + 4 = 8: Cancers falling into this category typically consist solely of poorly formed, fused, and/or cribriform glands. Accurately assigning the appropriate Gleason score to a diagnosed prostate cancer is an essential undertaking. Furthermore, a discussion persists among experts, with some questioning the classification of Gleason 6 as a form of cancer. Several urologists view Gleason 6 as a benign growth with the capacity for invasiveness, yet unlikely to metastasize to other organs (3).

Another important factors in diagnosing PCa are total prostate-specific antigen (tPSA), free PSA (fPSA), PSA density (PSAD), and the free-to-total PSA ratio (f/t PSA). They are well-established clinical markers for detecting and grading PCa (4). Nonetheless, there remains a lack of consensus concerning which indicators are most suitable for the diagnosis and grading of PCa. The clinical utility of these markers is hindered by certain limitations, including the issue of overdiagnosis and subsequent overtreatment. Consequently, there is an urgent requirement for a novel approach to early and precise risk stratification of PCa to ensure favorable prognoses for patients.

Over the past few years, there has been a growing utilization of multi-parameter MRI (mp-MRI) in localizing, qualitatively assessing, and diagnosing staging of PCa (5). The aim of PI-RADS is to enhance the consistency of prostate MRI examination and analysis. PI-RADS version 2.1 undertones the role of contrast enhancements suggesting the implementation of biparametric MRI (bp-MRI), which involves utilizing only T2WI and DWI sequences, to streamline the process of prostate MRI (6). Radiomics, a promising and trending field of research, utilizes MRI to assess tumor heterogeneity and has demonstrated good diagnostic efficacy. It involves the extraction of a vast number of imaging features in a high-throughput manner, which are subsequently transformed into mineable high-dimensional data. Through quantitative analysis of this data, radiomics presents an unparalleled occasion to enhance clinical decision-making (7).

Machine Learning (ML) techniques are specifically crafted to analyze vast quantities of high-dimensional data, without relying on specific biomedical hypotheses, with the aim of directly uncovering actionable insights. As a result of these capabilities, ML methods, particularly those focused on classification, are progressively being integrated into radiomic investigations to enhance the assessment of PCa and mitigate subjectivity in the process (8).

In particular, by using ML to develop a model risk score based on MRI-directed pathway, clinical and demographic patient information could potentially achieve a more favorable equilibrium between the dangers associated with biopsy-related complications and the possibility of over diagnosing PCa, while also mitigating the risk of overlooking clinically significant prostate cancer (9).

The selection of patients for prostate biopsy, who are suspected of having clinically significant prostate cancer, continues to pose a challenge, despite the increasing array of diagnostic resources (10). The current diagnostic tools mainly rely on PSA, employing various forms of PSA, alternative molecular markers, or combinations of these markers (11). Numerous tools have been devised to anticipate low-risk PCa by leveraging clinical parameters, including clinical T-stage, PSA, PSAD, prostate volume, prostate, and patient age (10, 12, 13). However, their low specificity leads to over diagnosis. Only a few studies as reported in a recent review (14) integrated an MRI score to the model by achieving a higher accuracy.

Therefore, the main aim of this study is to 1) automatically characterize PCa patients to avoid unnecessary biopsy; 2) identify a risk score to help stratify clinically insignificant PCa.

The novelty of this proof-of-concept study is to investigate multicentric bi-parametric MRI features (no contrast agents) along with clinical and demographic information to inform on unnecessary biopsy.

However, to the best of our knowledge, no attempts have been made to utilize radiomics from bi-parametric MRI for developing a risk score model. Although this is a piloting study and more advanced analysis on a bigger cohort are necessary to overcome some of the study limitations.

## 2 Materials and methods

### 2.1 Datasets

Data were acquired from two centers. Patient imaging and histopathology records were collected from H.S. Maria delle Grazie, Italy, and H.S. Maria di Terni, Italy. For the first center, data from 135 patients were included in this study who underwent prostate MRI between April 2013 and September 2018 due to elevated PSA levels and/or clinical suspicion of PCa, subsequently followed by biopsy. In the case of the second center, this retrospective study included 54 patients who had undergone prostate MRI between July 2015 and October 2021 due to heightened PSA levels and/or clinical suspicion of PCa, leading to subsequent biopsies. Biopsy outcomes were deemed the benchmark in both centers, specifically categorizing lesions as positive for PCa in instances where GS ≥ 4. Clinically insignificant lesions were considered with GS=3 + 3. Further information on the dataset can be found in Brancato et al. (15) and Castaldo et al. (16).

### 2.2 Image e acquisition and biopsy protocol

In the case of center 1, the MRI acquisition protocol entailed the capture of T2W, T1W, DCE-MRI, and DWI images (b values of 50, 400, and 1000 s/mm^2^), including an ADC map generated during the imaging procedure. Imaging was performed using a MAGNETOM-Avanto scanner (Siemens Healthcare, Erlangen, Germany) operating at 1.5 T. As for center 2, the MRI acquisition involved the acquisition of T2W and T1W images, along with DWI (b value ranging from 0 to 2000 s/mm2), including an ADC map. Patients were examined using a MAGNETOM-Verio scanner (Siemens Healthcare, Erlangen, Germany) operating at 3 T. The comprehensive technical parameters of the MRI sequences can be found in Castaldo et al. (16).

All biopsies of the prostate were guided by TRUS and conducted with anesthesia, utilizing an 18-gauge Tru-Cut needle subsequent to an imaging procedure. A highly experienced senior pathologist, unaware of the MRI findings and with more than 10 years of expertise in analyzing prostate samples, assessed the pathological sections. Tumor categorization was based on the 4th WHO classification, with additional grading determined by the Gleason score (GS) and the cancer group grade. Further details are reported in Castaldo et al. (16).

### 2.3 Image processing and radiomics feature extraction

Two skilled radiologists collaborated in outlining 3D regions of interest (ROIs) in the suspected lesions through consensus, while inspecting the b = 1000 (for center 1) and b = 1500 (for center 2) DWI volume. Two experienced radiologists were asked to draw 3D regions of interest (ROIs) in the suspected lesions in consensus while also looking at the b = 1000 (for center 1) and b = 1500 (for center 2) DWI volume. In particular, radiologists A and B focused on drawing ROIs in the suspected lesions according to the previous criteria, while radiologist B provided support by cross-verifying the ROIs and ensuring consensus was reached. Any discrepancies or uncertainties were discussed, and consensus was reached through mutual agreement. During the segmentation process, the radiologists remained unaware of the histology results and any clinical information associated with the retrospective prostate MR images. Prior to the extraction of radiomic features, the normalization of T2W image intensities was implemented. B-spline interpolation was employed to rectify variances stemming from parameters related to voxel size and to standardize the voxel size throughout the cohort. Following recommendations from the PyRadiomics community, each image was discretized by resampling the grayscale values utilizing a fixed bin width, enabling the acquisition of an optimal bin count within the range of 16–128. A total of 196 radiomics features were extracted using the open-source Python package PyRadiomics (https://pyradiomics.readthedocs.io/en/latest/, accessed on 1 July 2022) for T2W and ADC sequences. The first order and multidimensional texture features were amalgamated and denoted as “original features.” Further particulars are outlined in Castaldo et al. (16).

### 2.4 Framework of analysis and statistical analysis

**Figure 1:**
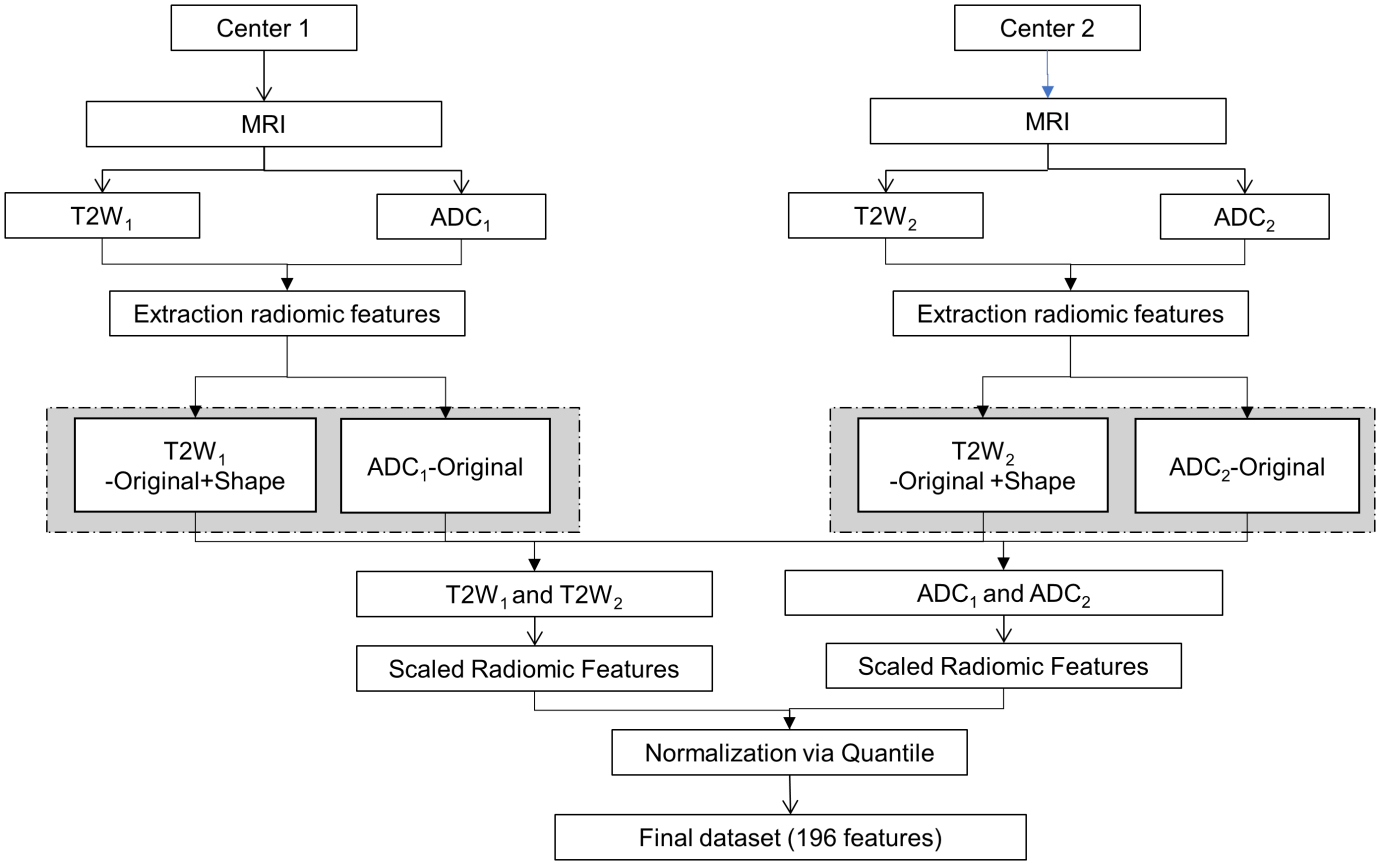
Framework of analysis. MRI, Magnetic Resonance Imaging; T2W, T2-weighted.

The framework for the proposed analysis is illustrated in **Figure 1**. MRI images were obtained using distinct acquisition protocols from centers 1 and 2. Specifically, the exploration encompassed T2W and ADC images, with the extraction of radiomic features aiming to investigate the variability of radiomics and pinpoint the sources of variability in the multicenter study.

To minimize any potential radiomics variability, image post-processing techniques were employed, and batch effects were scrutinized. Initially, the extracted radiomic features were scaled. Each radiomic feature underwent centering and scaling utilizing the generic function in R: scale. This process aimed to reduce inherent discrepancies in their scale and range (16, 17). Subsequently, the scaled radiomic features underwent further normalization through a quantile normalization method. The technique of quantile normalization transforms the initial data, eliminating undesired technical variation by enforcing the observed distributions to align with the average distribution, derived from averaging each quantile across the samples (18). Batch effects were investigated for both T2W and ADC radiomic features and across modalities, but batch effects were not visible and therefore, none of the methods described in Castaldo et al. (16) were applied.

### 2.5 Risk model approach

**Figure 2:**
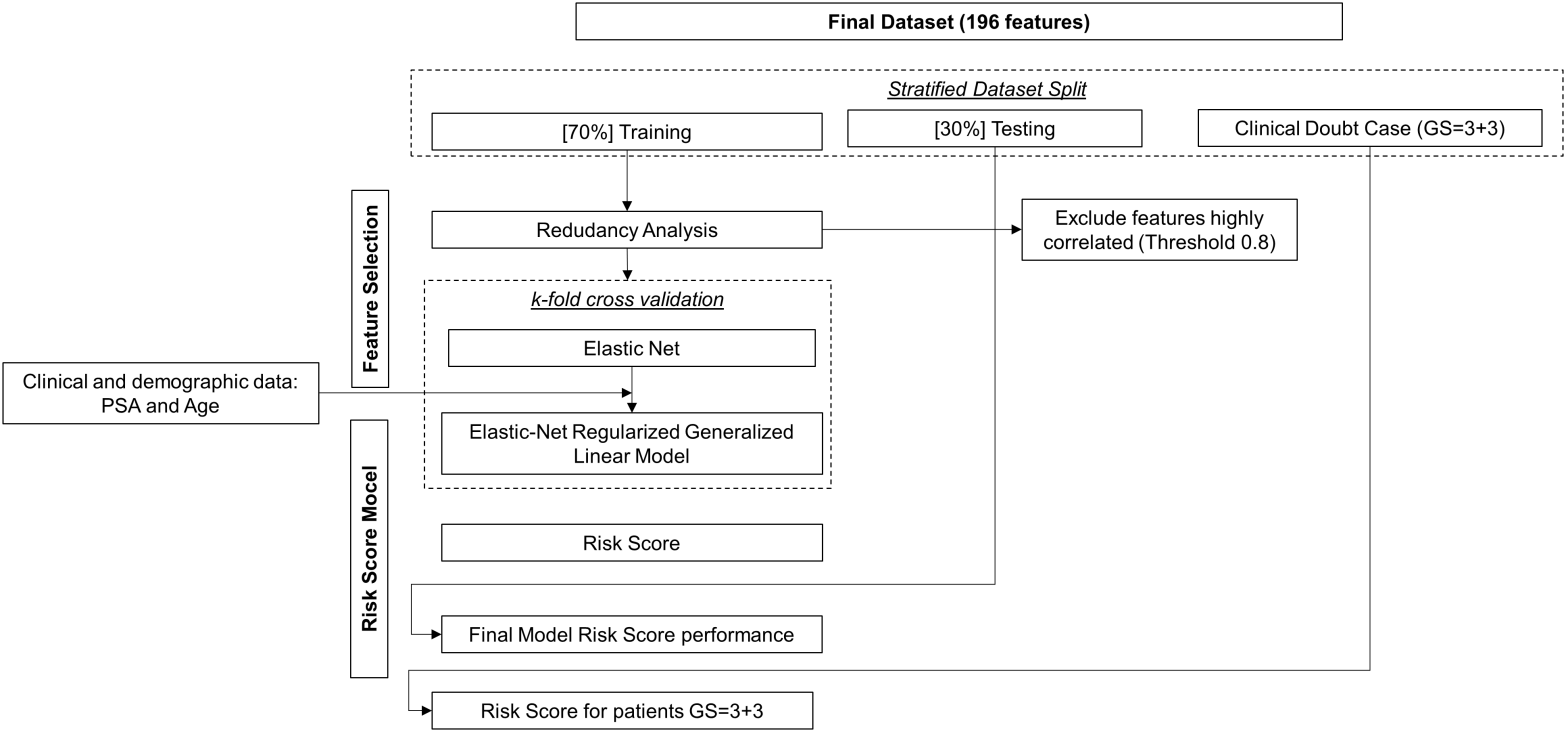
Model Risk Score.

The proposed framework used to develop a risk model score is shown in **Figure 2**. The final dataset was split into clinically significant PCa (GS≥4), non-clinically Significant PCa (GS ≤ 3) and clinically insignificant PCa (GS=3 + 3). For the first part of the analysis, the patients with GS=3 + 3 were kept aside, and a binary classifier was developed. The data were stratified split in training (70%) and testing (30%). The patients with GS=3 + 3 were used to assess the risk score according to their bi-parametric PI-RADS.

#### 2.5.1 Feature selection

Due to the extensive quantity of features, the process of feature selection was deemed a crucial stage in constructing a resilient model. Indeed, the final classifier’s feature count and dimensionality needed to be constrained in accordance with the number of subjects manifesting the event being detected, thus mitigating the risk of overfitting in the machine learning model. Additionally, a concise selection of clinical features significantly streamlines the clinical interpretation of the results, directing focus solely on the most informative and pertinent radiomic and clinical features (19). Hence, the feature selection procedure was built on two primary steps: redundancy analysis (20) and Elastic Net based Feature Ranking (21). The redundancy analysis selected only one feature from each cluster of features mutually correlated using Spearman’s rank correlation to reduce multicollinearity in the model by using a threshold of │0.8│ and a p-value less of 0.05. The degree of association between the two variables is deemed highly robust when the coefficient falls within the range of 0.8 to 1, moderately strong between 0.6 to 0.7, weak between 0.3 to 0.5, and very feeble when below 0.2. When the correlation coefficient equals 0, it indicates that the two variables are entirely independent of each other (22–24).

Elastic Net based Feature Ranking, a method of regularized regression, introduces an L2 penalty in addition to circumvent the drawbacks associated with the least absolute shrinkage and selection operator (LASSO) (21). This technique estimates the feature weights and conducts feature selection concurrently, effectively assigning a weight of zero to most irrelevant features. The penalty parameters were tuned on the training set through a K-fold cross-validation. Features were ranked in a decreasing order based on their importance score.

#### 2.5.2 Training, validation and testing

The selected features were used to train and validate Elastic-Net Regularized Generalized Linear Model and develop a risk score (25).

We implemented the EN model utilizing the glmnet method within the “caret” package (26) in R (http://www.r-project.org/; release 3.6.0). The data underwent scaling and centring to zero (pre-process option in caret). During each iteration of the EN model training, a grid search was employed to fine-tune both α and λ.

Consequently, feature selection and the training of the machine-learning model (including the tuning of classifier parameters) were conducted using 70% of the total patient cohort. The training data was also utilized to validate the classifier through the implementation of a k-fold cross-validation technique. The model validation involved employing a 5-fold person-independent cross-validation approach. Subsequently, the model’s efficacy in automatically identifying clinically significant PCa patients was tested on an independent set of data (comprising approximately 30% of the total patient cohort). Binary classification performance metrics were adopted based on standard formulas (27).

#### 2.5.3 Model risk score

The “risk score” model was generated via the regression analysis and is expressed as a cut-off indicating the risk of significant PCa. The cut-off for the risk probability scores was established based on the test characteristics. Specifically, the cut-off was established as the median of the probability distribution plots within the test set. Moreover, the risk model score was also assessed against the bi-parametric PI-RADS v2.

## 3 Results

### 3.1 Study population

For this study, a total of 189 PCa patients were investigated (59 patients with positive biopsies, 73 patients with negative biopsies and 57 patients with GS=3 + 3). At H.S. Maria delle Grazie, Italy, 135 PCa patients were investigated (47 patients with positive biopsies, 50 patients with negative biopsies and 38 patients with GS 3 + 3); at H.S. Maria di Terni, Italy 54 PCa patients were investigated (12 patients with positive biopsies, 23 patients with negative biopsies and 19 patients with GS 3 + 3). The demographics and clinical characteristics of the study population are reported in **Table 1**.

**Table 1:** Study population.

	Positive Biopsy	Negative Biopsy	GS 3 + 3
**No. of patients (n (%))**	59 (31%)	73 (39%)	57 (30%)
**Median AGE (mean SD)**	69.362(6.809)	65.447(6.262)	66.280 (6.771)
**Mean PSA (ng (SD))**	14.092(15.010)	8.703 (6.258)	8.036 (6.095)
**Mean PSAD (ng/ml (SD))**	1.307 (6.973)	0.123 (0.09)	0.158 (0.211)
**Mean Prostate Volume (cm (SD))**	43.671 (20.295)	77.656 (39.958)	57.105 (27.163)

### 3.2 Classification risk model

The data were stratified split in 70% training (n=94) and 30% testing (n=38). The patients with GS=3 + 3 (N=57) were then assessed against the bi-parametric PI-RADS as external validation of the risk score.

#### 3.2.1 Feature selection

By using the training set, we started our feature significance analysis utilizing redundancy analysis and Elastic Net based Feature Ranking.

**Figure 3:**
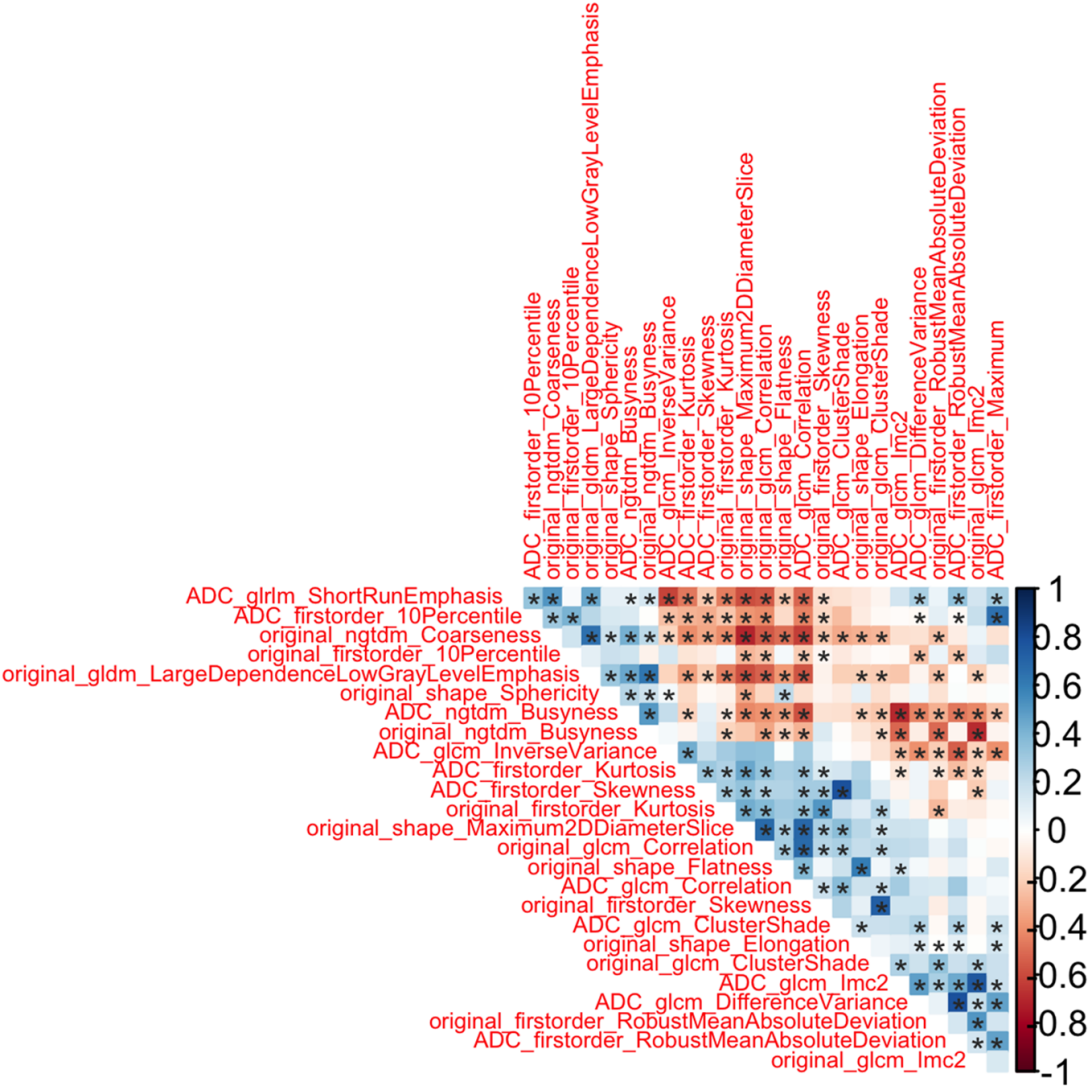
Correlation Plot as result of redundancy analysis *: p-value<0.05.

As first step, we performed redundancy analysis based on absolute values of Spearman correlation among statistically significant radiomic features. The features which were having lower correlation (with Spearman correlation below 0.8) have been labeled as redundant and removed from the final radiomic feature list. **Figure 3** showed that 26 radiomic features were not redundant.

**Figure 4:**
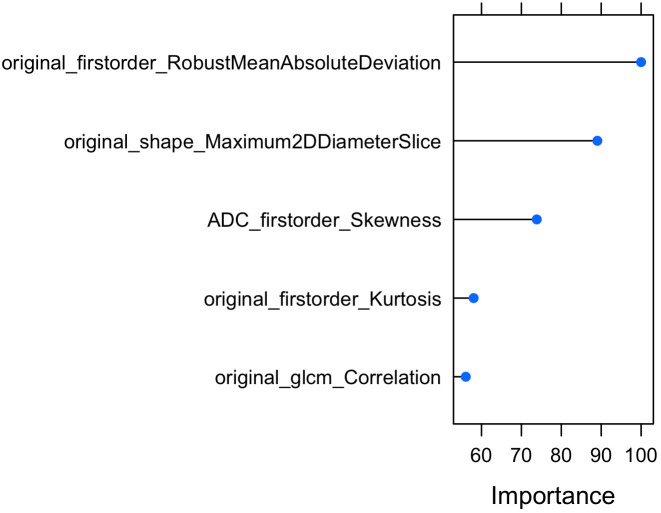
Elastic Net based Feature Ranking analysis (first 5 ranked features).

In the next step, Elastic Net based Feature Ranking was employed to further minimize the number of radiomic features. Elastic Net based Feature Ranking was applied to the training set and 5-fold cross validation was also employed. According to Elastic Net based Feature Ranking, we found that 17 radiomic features were ranked as possible predictors. According to Foster et al. (19), because of the limited number of patients in the study, no more than one feature was utilized for every ten observations or subjects manifesting the outcome of interest to construct the models, therefore, we choose the first 5 radiomic features (**Figure 4**).

In addition to the radiomic features selected by redundancy and elastic net, age and PSA were added as predictors to develop the model. Therefore, a total of 7 predictors were selected as input to the machine learning model.

#### 3.2.2 Training validation and testing

The model, Elastic-Net Regularized Generalized Linear Model, was trained and validated by using 5-fold cross validation techniques by using 7 predictors: original first order Robust Mean Absolute Deviation, original shape Maximum 2D Diameter Slice, ADC first order Skewness, original first order Kurtosis, original glcm Correlation, Age and PSA.

The hyperparameters of Elastic-Net Regularized Generalized Linear Model were tuned during the training and validation. The optimized parameters were alfa=0 and lambda=0.2.

**Figure 5:**
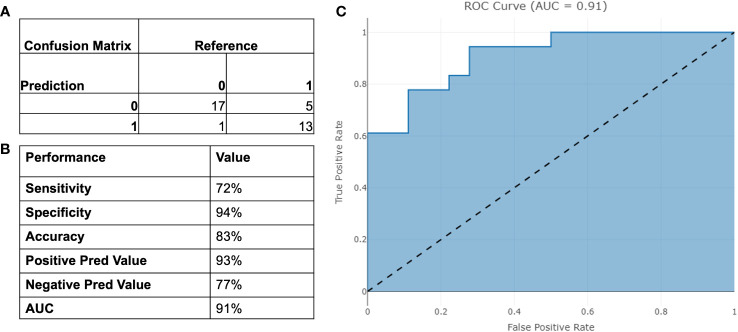
Performance of the binary model on testing set. **(A)** Confusion Matrix; **(B)** Binary Performance; **(C)** ROC curve.

The final model was then tested on a dependent set of data and performance are reported in **Figure 5**. The model achieved an AUC of 91% and an overall accuracy of 83% [95% CI 67%-94%].

#### 3.2.3 Model risk score

The model risk score is presented in the following equation:


$$\begin{array}{l} \text{y}∼2.61-0.49*\\ \;\text{original firstorder Robust Mean Absolute Deviation}+\\ \;0.35*\text{ original shape Maximum }2\text{D Diameter Slice}+0.19\\ \;*\text{ ADC firstorder Skewness}+0.24*\text{original firstorder Kurtosis}\\ \;+0.33*\text{ original glcm Correlation}+0.03*\text{Age}+0.02*\text{PSA} \end{array}$$

**Figure 6:**
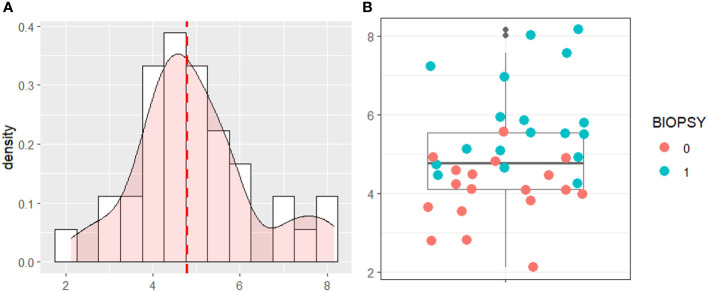
**(A)** Density Distribution of the Risk Score on the testing data. **(B)** Boxplot of the risk score with testing data.

The model risk score was plotted based on the testing data in **Figure 6**. The cut-off was chosen as the median of the distribution. The cut-off to automatically detect PCa patients was 4.769 [95%CI 4.483-5.18].

**Figure 7:**
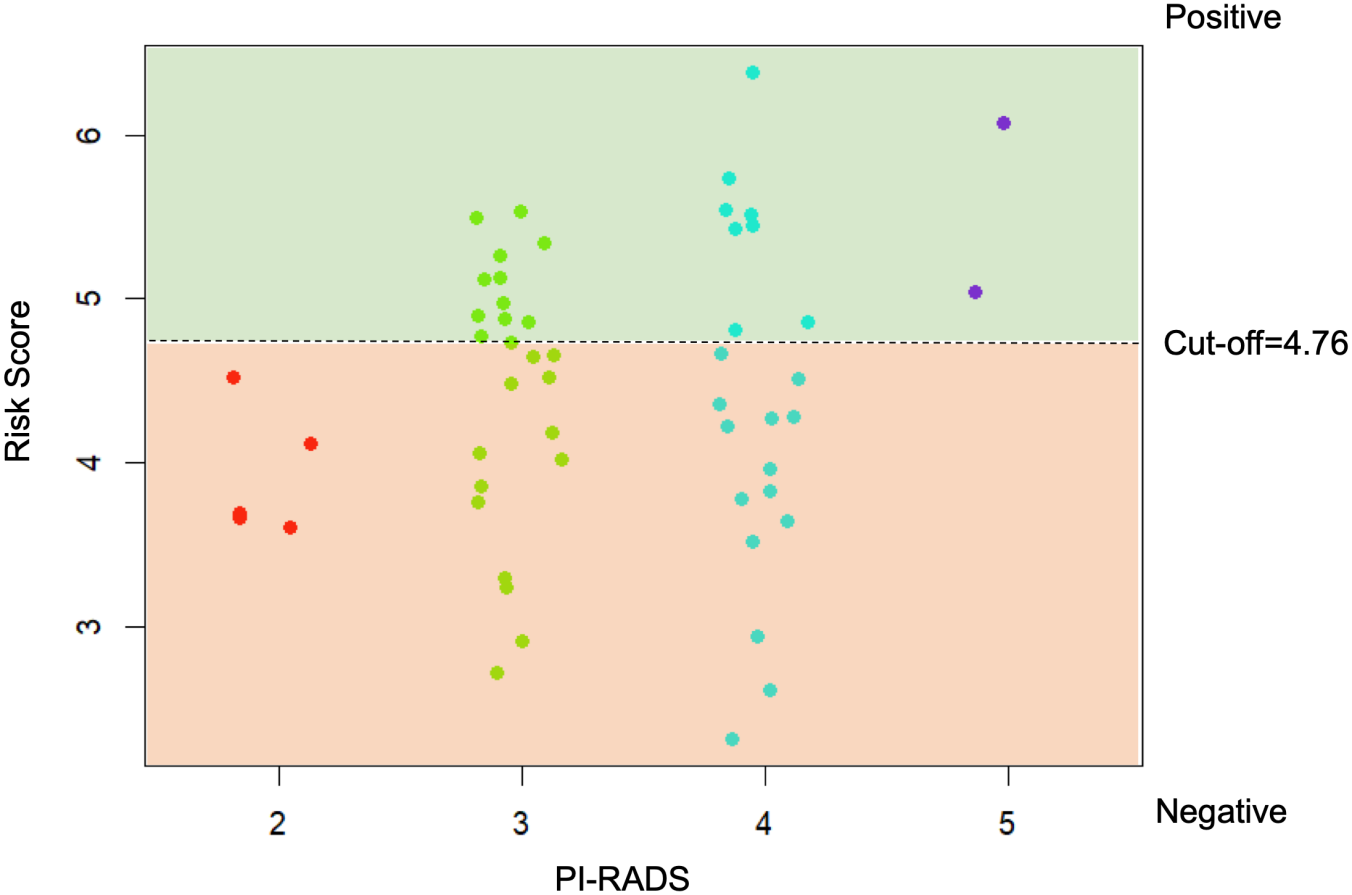
Risk Score on the clinically insignificant PCa with GS=3 + 3 against bi-parametric PIRADS.

The cases that were considered clinically insignificant PCa with GS=3 + 3 were used to assess the model risk score as shown in **Figure 7**. The proposed risk score was able to correctly identify patients (GS 3 + 3) with PI-RADS 2, which are considered at low risk to present a clinically significant cancer, and PI-RADS 5, in whose patients clinically significant cancer is highly likely to be present. For patients with PI-RADS 3 e 4, where clinically significant cancer is only likely to be present, the model risk score could be used to provide useful information on follow-up plans.

## 4 Discussion

Our study, consisting of 189 men from two different cohorts, shows that we can discriminate clinically significant PCa from other prostate conditions with a high specificity of 94% and 72% sensitivity. The model displays high NPV and PPV while limiting the number of missed cancers. The high NPV of 77% is particularly valuable, indicating that when the test predicts a negative result, there is a high level of confidence that significant disease is indeed absent. Similarly, the PPV of 93% provides useful insight into the likelihood of true positive results, aiding in clinical decision-making. Crucially, the model comprises solely of objectively identifiable input parameters, making it impervious to subjectively chosen parameters such a digital rectal exam (DRE), MRI suspicious score or prostate volume.

Other studies have also investigated risk scores to detect PCa patients by using molecular biomarkers and invasive measures. Strobl et al. (28) developed a static risk tool that predicts the likelihood of detecting prostate cancer if a prostate biopsy were to be performed. In fact, they used various factors associated with the patient, such as age, family history, prostate-specific antigen (PSA) levels, DRE results, and other relevant clinical information, which can also be quite invasive. One study, by Pye et al. (29), evaluated a novel diagnostic test, Proclarix, incorporating biomarkers like thrombospondin-1, cathepsin D, total and free PSA, and age for predicting clinically significant prostate cancer. They achieved a lower specificity of 43% but a higher sensitivity of 90% for detecting significant disease. This would mean that there might be some false positives (lower specificity), making the algorithm ineffective to reduce unnecessary prostate biopsies. Another study by Dutto et al. (12) also attempted the development of a novel risk score (NRS) incorporating various parameters such as PSA, prostate volume, age, clinical T Stage, and biopsy core data. By outperforming pre-existing predictive tools in both derivation and validation cohorts, the NRS demonstrates its potential for improved accuracy and reliability. However, Dutto et al. achieved a lower AUC value of 76% in the validation cohorts, whereas we achieved a 90% AUC leading to better patient stratification, facilitating more targeted and appropriate clinical interventions, thus potentially reducing both unnecessary treatments and missed diagnoses.

Few studies have investigated a risk score using imaging parameters and radiomics. A recent review (14) discusses the potential of personalized risk-based algorithms in prostate cancer diagnosis, utilizing predictive prebiopsy factors along with prostate-specific antigen. They showed that mpMRI as a secondary tool could enhance the detection of significant PCa. MRI risk models show variable performance in detecting clinically significant PCa, offering the ability to reduce biopsies and avoid detecting non-significant PCa, depending on chosen risk threshold. However, the studies used MRI suspicion scores, which are quantitative assessments used to evaluate the likelihood of prostate cancer based on the findings of a multi-parametric magnetic resonance imaging (mpMRI) scan. The area under the receiver-operating characteristic curve for detecting clinically significant PCa varies between 0.64 and 0.91 in biopsy-naïve men, and between 0.78 and 0.93 in men with a previous negative biopsy. However, to assess their wider applicability, in-depth analysis of mpMRI predictive qualities should be further investigated. For instance, Peters et al. (9) developed a risk score called Imperial RAPID risk score based on age, PSA density, prior negative biopsy, prostate volume, highest MRI score, which had good predictive ability. Adding family history, DRE, Black ethnicity to the eight-item score yielded similar results. RAPID score reduced biopsies while capturing significant cancers. However, they used an MRI score (PIRADS or Likert) that combines multiple imaging findings which can be subjective. Also, Sakaguchi et al. (13) retrospectively analyzed clinical parameters and bpMRI findings from 773 biopsy-naïve patients. They subsequently developed a risk model by employing a Multivariate logistic regression analysis to predict significant prostate cancer. The inclusion of parameters like age, log prostate-specific antigen (PSA), prostate volume, and PI-RADS scores in the risk model reinforced its comprehensiveness. However, also in this study they achieved a lower AUC (86%) for the risk model. In fact, our model indicates its superior discriminatory ability, underscoring its potential as a more reliable tool for ruling out cancer, while showing high sensitivity in cases where further confirmation is needed for suspected cases of prostate cancer. Hence, the existing risk stratification models, designed to estimate oncological outcomes, lack the capability to precisely outline the prognosis for individual patients across various stages of the disease. This underscores the persistent requirement for the development of personalized and precise detection tools and treatments.

In this study, we also tested our model risk score on clinically doubt patients that presented at biopsy a Gleason score of 3 + 3. Those patients are often associated with low-risk, slow-growing prostate cancer. Many men with this score opt for active surveillance. This means regular monitoring through PSA tests, digital rectal exams, and possibly repeat prostate biopsies. Our proposed risk assessment system effectively distinguished between patients with PI-RADS 2, indicating a low likelihood of clinically significant cancer, and PI-RADS 5, signifying a high likelihood of clinically significant cancer. For patients falling into the PI-RADS 3 and 4 categories, where the likelihood of clinically significant cancer is intermediate, the model’s risk score could offer valuable insights for follow-up plans. For example, patients identified as “negative” by the model’s risk score could be placed on a non-invasive and proactive surveillance regimen. This might involve regular monitoring, including periodic MRI scans and PSA tests, to track any changes in the lesion or prostate health. This approach could be pursued initially, eliminating the need for an immediate transrectal ultrasound or transperineal biopsy. Notably, the model demonstrated a high level of specificity in its overall performance.

Overall, the model under consideration in this study was developed using data obtained from MRI scans from two centers with various imaging protocols, including different settings or techniques used during the imaging process. The key point to note is that during these imaging procedures, no contrast agents were administered to the patients, thus reducing the invasiveness of the procedure. Furthermore, the effectiveness of the model’s risk category assignment, particularly concerning PI-RADS assessments, implies that it can accurately assess the risk category of a patient without the need for the administration of contrast medium. This is significant because the model’s performance is not dependent on the use of contrast agents, which can sometimes have associated risks or contraindications for certain patients.

However, there are some limitations. In our study, while we did not explicitly perform a feature repeatability test, but we implemented rigorous preprocessing steps to enhance the reliability of the extracted radiomic features. Firstly, we scaled the radiomic features to minimize differences in scale and range, which is essential for ensuring comparability across features. Subsequently, we further normalized the scaled features using quantile normalization to remove unwanted technical variation and ensure consistency across samples. Additionally, we investigated batch effects for both T2-weighted and ADC radiomic features, as well as across modalities, with no batch effects observed. This suggests that the preprocessing steps implemented in our study effectively mitigated potential sources of variability and ensured the robustness of the extracted radiomic features. However, incorporating a feature repeatability test in future studies could further strengthen the validity of our radiomic analyses (30).

Another possible limitation is the use of a multi-step feature selection process where potential errors or hurdles could have arose in the model such as: a) overfitting, in fact by choosing features solely based on the training set might result in overfitting, hence, we validated the chosen features using an independent test set; b) correlation challenges, in fact certain features may exhibit high correlation, leading to redundancy, consequently, we managed correlated features appropriately; and c) selection bias, in fact, improper execution of feature selection may introduce bias into the model, impacting its generalizability, therefore, we incorporated K-fold cross-validation as well.

Moreover, at present no follow-up data of patients presenting GS 3 + 3 and external validation set of data are available. There is, in fact, the need to calibrate these results in future studies. While our study included an internal validation in a multicenter context to gauge its broader applicability, we aim to scrutinize the model’s performance and make necessary adjustments based on newly acquired data. We advocate for additional validation on external cohorts comprising diverse patient populations with varying baseline risks, ensuring the risk prediction tool’s effective performance before its integration into clinical practice.

## 5 Conclusion

This study demonstrated that the integration of radiomic features, PSA, and age can achieve a significant level of clinical effectiveness in helping to determine the necessity of a prostate biopsy for individuals with suspected clinically significant PCa. The proposed methodology was scrutinized using a dataset encompassing 189 PCa patients from two medical centers. The Elastic-Net Regularized Generalized Linear Model achieved an impressive 91% Area Under the Curve (AUC) in automatically identifying PCa patients. This study explored the feasibility of a robust risk model to provide objective and precise risk stratification for PCa, which could inform personalized follow-up plans. Ongoing multicenter clinical studies will expand on these results in additional cohorts and assess how this test could support the clinicians.

## Data availability statement

The original contributions presented in the study are included in the article/supplementary material. Further inquiries can be directed to the corresponding author.

## Ethics statement

The studies involving humans were approved by Clinical Research and Ethical Review Board of the Regional Ethics Committee of Umbria (protocol number 25069/22/ON) and ethics committee of the Istituto Nazionale Tumori “Fondazione G. Pascale” (protocol number 1/20). The studies were conducted in accordance with the local legislation and institutional requirements. The participants provided their written informed consent to participate in this study.

## Author contributions

RC: Conceptualization, Formal analysis, Investigation, Methodology, Writing – original draft, Writing – review & editing. VB: Data curation, Formal analysis, Investigation, Writing – review & editing. CC: Conceptualization, Data curation, Investigation, Supervision, Visualization, Writing – original draft, Writing – review & editing. LP: Investigation, Methodology, Validation, Writing – review & editing. EI: Data curation, Resources, Writing – review & editing. EC: Conceptualization, Data curation, Resources, Writing – review & editing. AR: Conceptualization, Data curation, Resources, Writing – review & editing. MS: Conceptualization, Funding acquisition, Investigation, Project administration, Resources, Writing – review & editing. EN: Conceptualization, Data curation, Funding acquisition, Investigation, Methodology, Project administration, Resources, Writing – review & editing. MF: Conceptualization, Data curation, Investigation, Methodology, Project administration, Supervision, Writing – original draft, Writing – review & editing.
